# Two-Way Bending Properties of Shape Memory Composite with SMA and SMP

**DOI:** 10.3390/ma2031180

**Published:** 2009-09-01

**Authors:** Hisaaki Tobushi, Shunichi Hayashi, Yoshiki Sugimoto, Kousuke Date

**Affiliations:** 1 Department of Mechanical Engineering, Aichi Institute of Technology / 1247 Yachigusa, Yakusa-cho, Toyota, 470-0392, Japan; 2 SMP Technologies Inc./Shendagaya, 3-50-11, Shibuyaku, Tokyo, 151-0051, Japan; E-Mail: hayashi@smptechno.com (S.H.); 3 Citizen Machinery, Co., Ltd./4107-6 Miyota, Kitasaku, Nagano, 389-0206, Japan; E-Mail: pp08715@aitech.ac.jp (Y.S.)

**Keywords:** shape memory alloy, shape memory polymer, shape memory effect, superelasticity, composite, two-way deformation, bending, recovery force

## Abstract

A shape memory composite (SMC) was fabricated with a shape memory alloy (SMA) and a shape memory polymer (SMP), and its two-way bending deformation and recovery force were investigated. The results obtained can be summarized as follows: (1) two kinds of SMA tapes which show the shape memory effect (SME) and superelasticity (SE) were heat-treated to memorize the round shape. The shape-memorized round SMA tapes were arranged facing in the opposite directions and were sandwiched between the SMP sheets. The SMC belt can be fabricated by using the appropriate factors: the number of SMP sheets, the pressing force, the heating temperature and the hold time. (2) The two-way bending deformation with an angle of 56 degrees in the fabricated SMC belt is observed based on the SME and SE of the SMA tapes during heating and cooling. (3) If the SMC belt is heated and cooled by keeping the bent form, the recovery force increases during heating and degreases during cooling based on the two-way properties of the SMC. (4) The development and application of high-functional SMCs are expected by the combination of the SMA and the SMP with various kinds of phase transformation temperatures, volume fractions, configurations and heating-cooling rates.

## 1. Introduction 

The intelligent materials, which respond to optical, electrical or thermal actions from outside and perform various kinds of high function, have been developed and have attracted worldwide attention. Metals, ceramics and polymers have been developed as intelligent materials and applied widely to the fields of industry, aerospace, medical treatment and the products related to daily life. One of the main materials which have developed this research and applications are shape memory alloys (SMAs) [1,2,3]. Shape memory polymers (SMPs) have also been used practically [4,5,6].

In the SMA, the shape memory property appears based on the martensitic transformation (MT) in which the crystal structure varies depending on the variation in temperature. In the SMA, a strain of 8% is recoverable and high recovery stress can be used. The properties of energy storage and dissipation can be also used [7]. Among the SMA, the TiNi SMA shows excellent fatigue strength [8].

In the SMP, the shape memory property appears based on the glass transition in which the characteristics of molecular motion vary depending on the variation in temperature. In the SMP, sheet, film, foam and other forms can be used, and the strain of several hundred percents is recoverable. In the SMP, the polyurethane SMP has been used practically. Compared with SMAs, SMPs are light, transparent, easy to form and cheap. However, the recovery stress and fatigue strength are inferior to the SMAs [9].

In order to use new and higher functions by combining the excellent qualities of both SMAs and SMPs, the development of a shape memory composite (SMC) incorporating SMA and SMP is possible. If the SMP is used as the matrix in the SMC and the SMA as the fiber, the following properties can be obtained in the SMC: in the SMA, high recovery stress is obtained at high temperature and the residual strain which appears at low temperature disappears by heating. However, both the MT yield stress and elastic modulus are low at low temperature and therefore it is hard to carry a large load. On the other hand, the SMP is soft at high temperature and its original shape is recovered during unloading. The elastic modulus is high at low temperature and the SMP element can carry a large load by keeping the deformed shape. Therefore, if a SMC which combines the characteristics of both the SMA and the SMP is developed, (1) a large recovery force appears at high temperature and (2) the deformed shape is recovered, and (3) the deformed shape is held at low temperature and (4) a large load can be carried. Combining the SMA and the SMP, a shape memory element can be developed, in which the response speed is high and the recovery shape is prescribed precisely [10].

In the present paper, the fabrication method and the mechanical properties of a SMC which shows the two-way deformation depending on temperature variation are investigated. As the first step to develop the SMC, a SMC belt combined with two kinds of SMA and SMP is fabricated on an experimental basis, and the fabrication method of the two-way SMC and the basic mechanical properties of the SMC are investigated. With respect to the working characteristics of the SMC belt, the bending deformation and the recovery force with the two-way property by heating and cooling in the three-point bending test are discussed. A simple model is applied in the analysis of the two-way deformation and recovery force for the SMC belt.

## 2. Characteristics of the SMC with SMA and SMP

**Figure 1:**
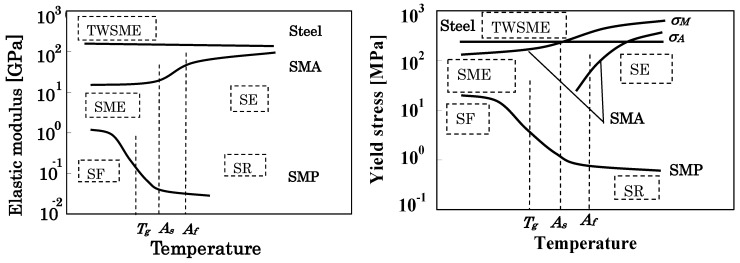
Dependence of elastic modulus and yield stress on temperature for SMA, SMP and steel.

In order to discuss the basic deformation properties of the SMA and SMP, and the characteristics of the SMC with SMA and SMP, the dependence of the elastic modulus and the yield stress of the SMA, the SMP and the steel on temperature is shown in Figure 1(a) and (b), respectively. Figure 1 is expressed on the semi-logarithmic graph.

In Figure 1, *A_s_*, *A_f_* and *T_g_* on the temperature axis denote the reverse (leading to austenite phase) transformation start and finish temperatures of the SMA and the glass transition temperature of the SMP, respectively. The symbols *σ_M_* and *σ_A_* represent the stress-induced MT stress and reverse transformation stress, respectively.

As shown in Figure 1, the elastic modulus and *σ_M_* are small at temperatures below *A_s_* and large at temperatures above *A_f_* for the SMA. The reverse transformation stress *σ_A_* appears around *A_f_*. Both *σ_M_* and σ*_A_* increase in proportion to the temperature. Based on these characteristics, if the SMA is deformed below *A_f_*_,_ the residual strain appears after unloading and the residual strain disappears by heating under no load, showing the shape memory effect (SME). If the SMA is deformed above *A_f_*, strain is recovered during unloading, showing the superelasticity (SE). On the other hand, the elastic modulus and the yield stress of the steel are almost constant in the temperature range of 273 K–373 K at which most SMAs have been practically used. Therefore, if the steel is used as a bias element in combination with an SMA element in the temperature region above and below *A_f_*, the two-way shape memory effect (TWSME) can be achieved by heating and cooling.

On the other hand, the elastic modulus and the yield stress of the SMP are large at temperatures below the glass transition temperature *T_g_* and small at temperatures above *T_g_*. Therefore, the SMP is easily deformed above *T_g_*. If the SMP is cooled down to the temperature below *T_g_* by holding the formerly deformed shape constant, the deformed shape is fixed and the SMP can carry large load. This property is called the shape fixity (SF). If the shape-fixed SMP element is heated up to the temperature above *T_g_* under no load, the original shape is recovered. This property is called the shape recovery (SR).

As mentioned above, the dependence of the elastic modulus and the yield stress on temperature is quite different between the SMA, the SMP and the steel. Therefore, if the composite material is produced by combining these materials appropriately, the SMC with new functions which are not possible with the component materials can be developed. For example, though the rigidity of the SMP element is low at high temperature, the rigidity of the SMC elements combined with the SMA becomes high at high temperature and large recovery force can be obtained simultaneously. In the similar way, though the rigidity of the SMA element is low at low temperature, the rigidity of the SMC element combined with the SMP becomes high at low temperature.

## 3. Fabrication of SMC Belt with Two-Way Movement

### 3.1. Materials

With respect to the SMA, two kinds of SMA tapes showing SME and SE at room temperature were used. The SMA tape showing SME was a TiNi SMA tape with a width of 5 mm and a thickness of 0.25 mm produced by Furukawa Techno Material Co. The SMA tape showing SE was a TiNi SMA tape with a width of 2.5 mm and a thickness of 0.3 mm produced by Yoshimi Inc. In the shape memory processing, each SMA tape was set along a fixing circular ring with an inner diameter of 16 mm and was heat-treated to memorize the round shape with an outside diameter of 16 mm. The reverse-transformation finish temperature *A_f_* of the SMA tape showing SME was 347 K and that showing SE was 317 K. With respect to the SMP, a polyurethane SMP sheet (MM6520) produced by DiAPLEX Co., Ltd. was used. Thickness was 0.25 mm and the glass transition temperature *T_g_* was 338 K.

### 3.2. Structure and Deformation Properties of SMC Belt

The SMC belt with a length of 60 mm, a width of 10 mm and a thickness of 0.75 mm was fabricated by using two kinds of SMA tapes and the SMP sheet. In the fabricated SMC belt, the SMP sheet was used as a matrix and the SMA tape as a fiber. Length, width and thickness of the SMA tape showing SME (SME-SMA tape) were 50 mm, 5 mm and 0.25 mm, respectively. Length, width and thickness of the SMA tape showing SE (SE-SMA tape) were 50 mm, 2.5 mm and 0.3 mm, respectively. The two kinds of SMA tapes were located in the central part of the SMC belt. The area fraction of the SMAs in cross-section was 27% in the fabricated SMC belt.

**Figure 2:**
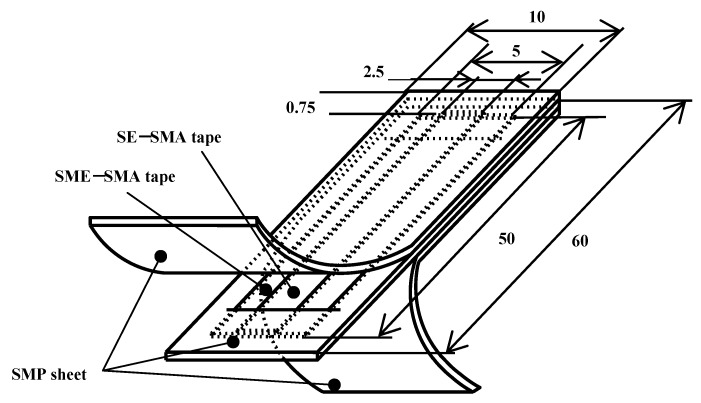
Structure of SMC composed of SME-SMA tape, SE-SMA tape and SMP sheet.

**Figure 3:**
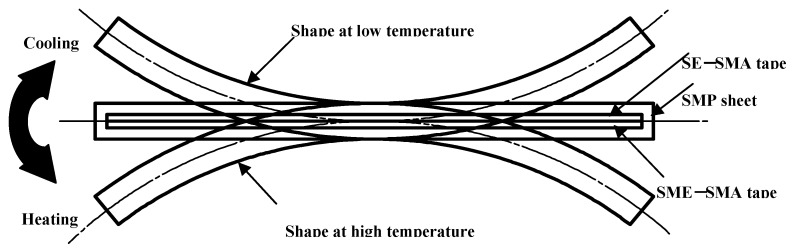
Principle of two-way bending behavior in SMC during heating and cooling.

The structure of the SMC belt is shown in Figure 2. As seen in the Figure, the two kinds of shape-memorized round SMA tapes were arranged facing in the opposite directions. The principle of the two-way bending behavior in the SMC belt during heating and cooling is shown in Figure 3. In Figure 3, the SMC belt is bent convexly downward (in the direction of the memorized round shape of the SE-SMA tape) by the recovery force of the SE-SMA tape at low temperature. It bends convexly upward (in the direction of the memorized round shape of the SME-SMA tape) by recovery force of the SME-SMA tape at high temperature.

### 3.3. Fabrication of SMC Belt

**Figure 4:**
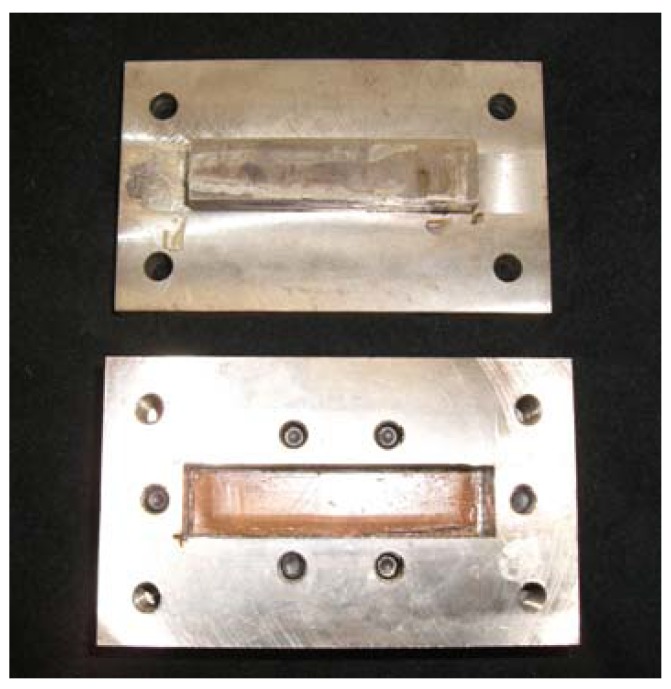
Photographs of the mold for fabricating the SMC belt.

**Figure 5:**
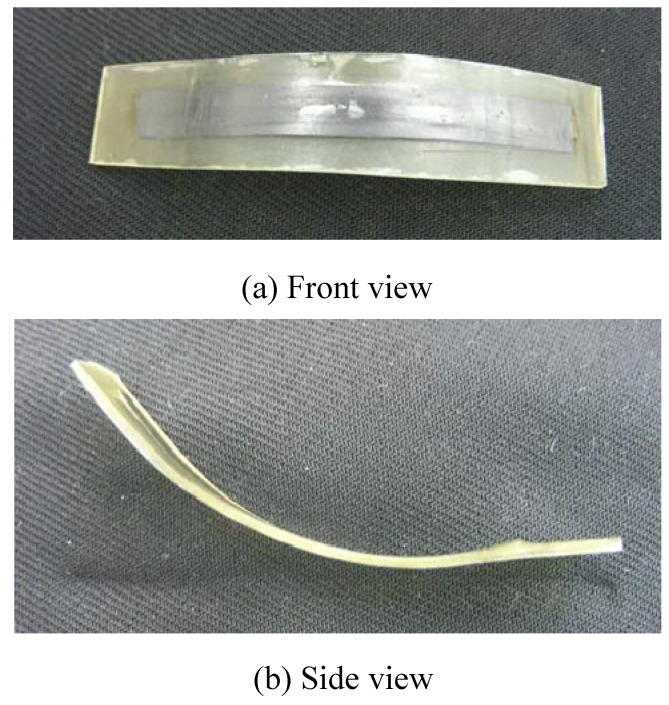
Photographs of the fabricated SMC belt.

At first, two cuts per tooth were made on one SMP sheet and the two kinds of SMA tapes (SME-SMA tape and SE-SMA tape) were passed through these cuts. In this process, the SME-SMA tape and the SE-SMA tape were arranged facing in the opposite directions for the memorized round shape. The SMP sheet passed through the two kinds of SMA tapes was sandwiched between two SMP sheets from upper and lower sides. The combined material was set in the mold shown in Figure 4 for fabricating the SMC belt. The fabricating conditions: the number of SMP sheets, the pressing force, the heating temperature and the holding time, affect the performance of the fabricated SMC belt. With respect to these factors, many preparatory experiments were carried out and the appropriate fabricating conditions were examined. As the result, it was confirmed that an SMC belt without bubbles and gaps between materials could be fabricated under the following conditions: first, two kinds of SMA tapes were sandwiched between the SMP sheets and set in the mold. Next, the upper and lower molds were fastened through the bolts by a torque of 6.78 N·m. The mold was held in the furnace at 448 K for 30 min followed by cooling in air. The photographs of the fabricated SMC belt are shown in Figure 5. As seen from the Figure, a small built-up part appeared near the edge of the SMA tape. In the case of one SMP sheet, the edge of the SME-SMA tape projected by the recovery force appeared from the surface during heating. In order to protect the projection of the edge, a small piece of the SMP sheet was put on the edge of the SME-SMA tape and the SMC belt was fabricated. The projection of the edge of the SME-SMA tape during heating was protected.

## 4. Two-Way Bending Deformation of SMC Belt

### 4.1. Two-Way Deformation Behavior

**Figure 6:**
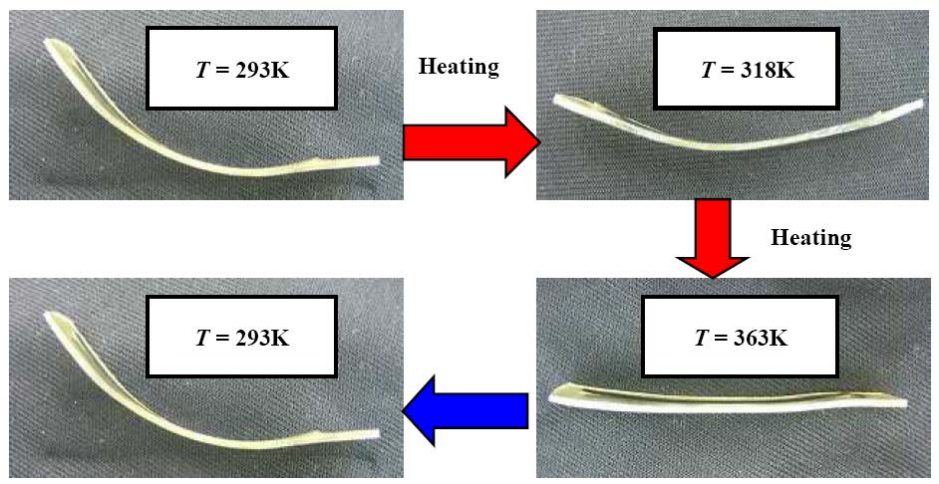
Photographs of the two-way bending deformation of the SMC belt during heating and cooling.

A photograph of the two-way bending deformation of the fabricated SMC belt during heating and cooling is shown in Figure 6. The heating and cooling were carried out between 293 K and 363 K. At 293 K, the force induced in the SE-SMA tape is high, and therefore the SMC belt bends in the direction of the shape-memorized round shape of the SE-SMA tape. If the SMC belt is heated, the SMP becomes soft and recovery force in the SME-SMA tape increases, and therefore the SMC belt bends in the direction of the shape-memorized round shape of the SME-SMA tape, resulting in the flat plane at 363 K. If the SMC belt is cooled thereafter, the recovery force in the SME-SMA tape decreases and that in the SE-SMA tape becomes higher. Therefore, the SMC belt bends again to the original shape at 293 K. The fabricated SMC belt bends in two directions by an angle of 56° during heating and cooling.

### 4.2. Evaluation of Deflection Based on Internal Bending Moment

The two-way bending deformation due to variation in temperature appears based on the internal bending moment induced in the SE-SMA tape, the SME-SMA tape and the SMP sheet of the SMC belt. The internal bending moment is proportional to the bending rigidity of each element and its dependence on temperature can be evaluated as follows. The bending rigidity of the strip is expressed by *EI* where *E* denotes the elastic modulus and *I* the second moment of area. If the width of cross-section in the strip is *b* and the height *h*, *I* = *bh*^3^/12. The bending rigidity of the SMC belt *E_c_I_c_* is given by a sum of the bending rigidity in each element as follows:

(1) $$E_{C}I_{C}=E_{SE}I_{SE}+E_{SME}I_{SME}+E_{P}I_{P}$$

where *E_c_*, *E_SE_*, *E_SME_* and *E_p_* denote the elastic modulus of the SMC belt, the SE-SMA tape, the SME-SMA tape and the SMP sheet, respectively. *I_c_*, *I_SE_*, *I_SME_* and *I_p_* represent the second moment of area of the SMC belt, the SE-SMA tape, the SME-SMA tape and the SMP sheet, respectively. There is a following relation:

(2) $$I_{C}=I_{SE}+I_{SME}+I_{P}$$

The bending rigidity of the SMC belt is given by the sum of the bending rigidity of each element as expressed by Equation (1). However, in the case of the phase transformation by heating and cooling under no load, the internal bending moments in the SMA elements, which are proportional to the bending rigidity of the SE-SMA tape *E_SE_I_SE_* and that of the SME-SMA tape *E_SME_I_SME_*, act to bend the SMC belt in each direction of the shape-memorized round shape. The internal bending moment in the SMP element, which is proportional to the bending rigidity of the SMP sheet *E_P_I_P_*, acts to bend the SMC belt in the direction of shape-memorized flat shape.

With respect to evaluation of the second moment of area obtained from Equation (2), since the central SMP sheet sandwiched between the SE-SMA tape and the SME-SMA tape melts during holding the pressed state at 448 K in the fabricating process and therefore becomes very thin, it can be assumed that the neutral axis in the cross-section of the SMC belt coincides with the boundary face between the SE-SMA tape and the SME-SMA tape. Therefore, each second moment of area of the SMA elements is *I* = *bh*^3^/12 + *A*•(*h*/2)^2^ = *bh*^3^/3 where the area of the cross-section *A* = *bh*.

The elastic modulus of the TiNi SMA tape is 70 GPa at temperatures *T* > *A_f_* and 20 GPa at *T* < *A_s_*. Therefore, the bending rigidity of the SE-SMA tape *E_SE_I_SE_* is 1576 N•mm^2^ at temperatures above 293 K. *E_SME_I_SME_* of the SME-SMA tape is 520 N•mm^2^ at 293 K and 1842 N•mm^2^ at 363 K. The elastic modulus of the SMP sheet is 1 GPa at *T* < *T_g_* and 10 MPa at *T* > *T*_g_. Therefore, the bending rigidity of the SMP sheet *E_p_I_p_* is 301 N•mm^2^ at 293 K and 3 N•mm^2^ at 363 K. Based on the temperature dependence of the internal bending moment which is proportional to the bending rigidity for two SMA tapes and the SMP sheet, the SMC belt deforms as follows. At 293 K, the fabricated SMC belt is taken out from the mold, and the SMC belt bends in the direction of the shape-memorized round shape of the SE-SMA tape where the internal bending moment is largest. At 363 K, the SMC belt bends in the direction of the shape-memorized round shape of the SME-SMA tape where the internal bending moment is largest. The two-way deformation property of the SMC belt depends on the arrangement and fraction of the SMA and SMP elements and the rate of heating and cooling processes. For example, if the SMC is cooled rapidly, the SMP element located on the surface is cooled at first and the rigidity of the SMP increases, and therefore the shape at high temperature is fixed. From this fact, large recovery deformation can not be obtained by the force which appears in the SE-SMA element located inside of the SMC. In order to develop the SMC, it is necessary to clarify these properties.

## 5. Two-Way Recovery Force of SMC Belt in Bending

In applications of the SMC belt, we will use not only the bending deformation but also the recovery force during heating and cooling. In the present chapter, the two-way recovery force which appears in the fabricated SMC belt during heating and cooling will be discussed.

### 5.1. Experimental Method

**Figure 7:**
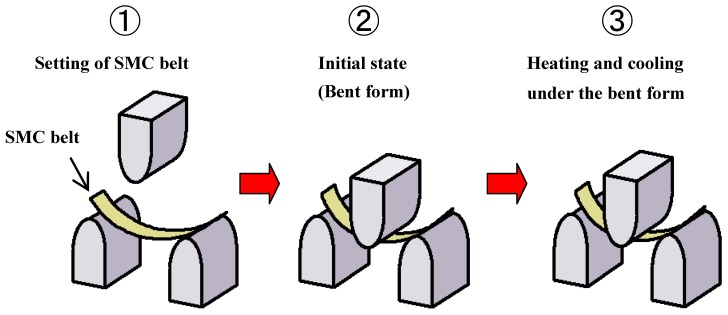
Experimental procedure of recovery force by the three-point bending test with heating and cooling.

The procedure of the three-point bending test with heating and cooling for the recovery force is schematically shown in Figure 7. At first, the bent-form SMC belt was set on the supports of the three-point bending test machine. The span was 30 mm. After setting the SMC belt, the point of the punch contacted the center of the SMC belt and the position was held constant. Keeping the initial bent form constant, the SMC belt was heated and cooled. The initial deflection between two supports under no-load was 3.4 mm before the cyclic thermal test.

**Figure 8:**
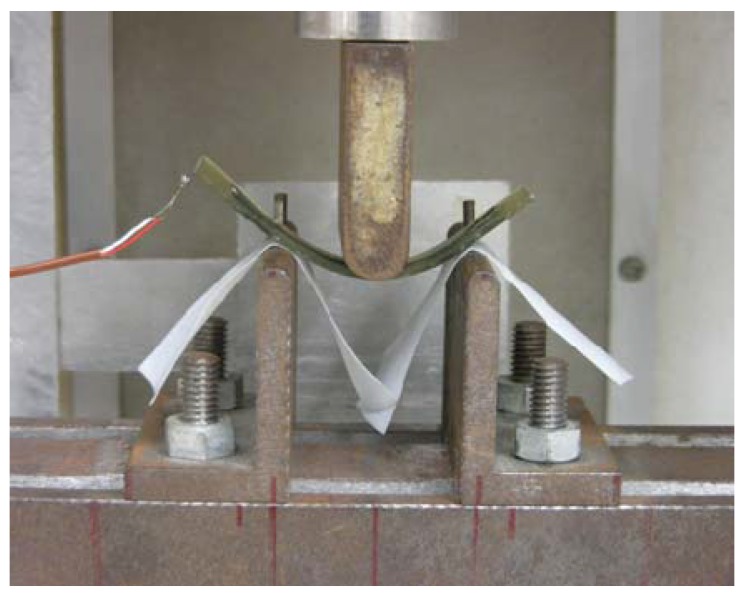
Photograph of the SMC belt kept in the bent form during heating and cooling in the three-point bending test.

The photograph of the SMC belt kept in the bent form during heating and cooling in the three-point bending test is shown in Figure 8. Heating and cooling rates in the furnace were about 1 K/min between 293 K and 363 K. The recovery force, which increases when the SMC belt tends to be flat plane during the heating process and decreases when the SMC belt tends to recover the original shape during the cooling process, was measured by a load cell.

### 5.2. Experimental Results and Discussion

**Figure 9:**
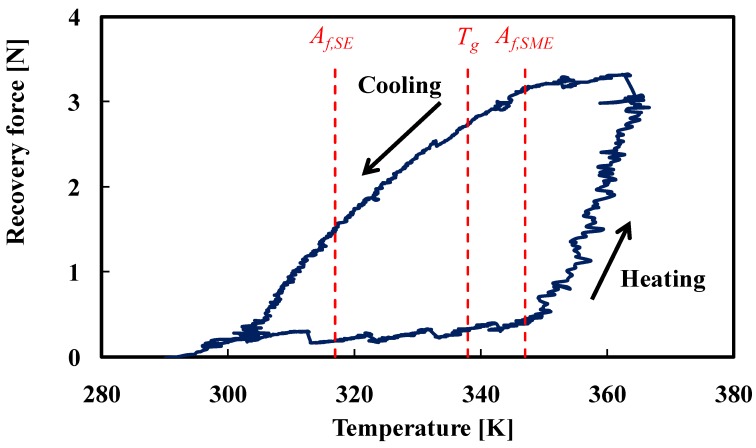
Relationship between recovery force and temperature during heating and cooling in the three-point bending test for the SMC belt.

The relationship between the recovery force and temperature obtained by the three-point bending test for the SMC belt is shown in Figure 9. The symbols *A_f_,_SE_*, *A_f_*,*_SME_* and *T_g_* shown in Figure 9 represent the reverse-transformation finish temperatures of the SE-SMA tape and the SME-SMA tape and the glass transition temperature of the SMP sheet, respectively. The behavior of the recovery force is different between the heating process and cooling process, and the curve describes a large hysteresis loop. In the heating process, since temperatures around the *A_f,SE_* of the SE-SMA tape are lower than *T_g_* of the SMP sheet, the elastic modulus of the SMP is high and therefore the rigidity of the SMP matrix is high. The recovery force of the SME-SMA tape does not appear at this temperature, and therefore the recovery force of the SMC is small. Although the elastic modulus of the SMP decreases at temperatures around *T_g_* of the SMP, the recovery force of the SME-SMA tape is smaller than that of the SE-SMA tape, and therefore the recovery force of the SMC belt is almost the same as that at temperatures around *A_f,SE_*. The recovery force starts to increase at temperatures around *A_f,SME_* of the SME-SMA tape. Since the recovery force of the SME-SMA tape becomes larger than that of the SE-SMA tape at temperatures around *A_f,SME_*, the recovery force of the SMC belt appears. In the whole heating process, the recovery force of the SME-SMA tape becomes larger than that of the SE-SMA tape at temperatures around 350 K, and therefore the recovery force of the SMC belt increases rapidly. 

The recovery force at high temperature can be evaluated as follows. The deflection *y* at the center of the SMC belt in the three-point bending is given by the following equation:

(3) $$y=\frac{Wl^{3}}{48E_{c}I_{c}}$$

where *W* and *l* denote the load applied at the center and the span, respectively. Therefore, the recovery force of the SMC belt at high temperature is obtained by the following equation:

(4) $$W=\frac{48E_{c}I_{c}}{l^{3}}y$$

The internal bending moment which appears in the SMC belt with the bent form at 363 K is as follows. Although the internal bending moment in the SME-SMA tape and the SMP sheet acts in the direction where the SMC belt returns to the flat plane, the internal bending moment in the SE-SMA tape acts in the opposite direction where the deflection increases. Therefore, from Equation (1), the bending rigidity of the SMC belt is obtained as *E_c_I_c_* = 251 N•mm^2^. Substituting the measured values of *l* = 25.9 mm and *y* = 3.4 mm in Equation (4), the recovery force *W* = 2.4 N. This value is 69% of the measured recovery force of 3.5 N at 363 K. Therefore, the recovery force induced the SMC belt can be roughly evaluated by Equation (4). The proposed simple model is useful in the analysis of the SMC.

In the cooling process, at temperatures around *A_f,SME_* of the SME-SMA tape, since the recovery force of the SME-SMA tape is larger than that of the SE-SMA tape, the recovery force of the SMC belt is high and almost constant. At temperatures around *T_g_* of the SMP, the recovery force of the SME-SMA tape is larger than that of the SE-SMA tape, the recovery force of the SMC belt is still high. At temperatures around *A_f,SE_* of the SE-SMA tape, though the elastic modulus of the SMP becomes large at temperatures below *T_g_*, the recovery force of the SMC belt is 1.5 N since the recovery force of the SME-SMA tape is still larger than that of the SE-SMA tape. In the whole cooling process, the recovery force of the SME-SMA tape decreases gradually and recovery force of the SMC belt decreases correspondingly.

The phenomenon in which the recovery force–temperature curve of the SMC belt describes a hysteresis loop during heating and cooling is similar to that in which the recovery stress–temperature curve of the SMA wire describes a hysteresis loop [11]. It is important to be noticed that the recovery force–temperature curve of the SMC belt depends on the *A_f_* points of the SMAs and *T_g_* of the SMP. The coefficient of thermal conductivity differs between the SMA and the SMP. Therefore, the characteristics of the deformation and the recovery force in the SMC depend on the arrangement and fraction of the SMA fiber and the SMC matrix and the rates of heating and cooling. These characteristics and the strength of the interface between the SMA and the SMP are the future subjects. The influence of the martenstic transformation of SMA on the behavior of the SMC in the cooling process is also the future subject.

## 6. Conclusions

The SMC belt composed of two kinds of the SMAs and the SMP was fabricated and the two-way deformation and the recovery force in bending were investigated. The results obtained can be summarized as follows:

(1) Two kinds of SMA tapes showing SME and the SE were heat-treated to memorize a round shape. The shape-memorized round tapes were arranged facing in the opposite directions and were sandwiched by one SMP sheet in the middle part and by two SMP sheets from upper and lower sides. The SMC belt was fabricated without bubble and gap by using the appropriate factors for the number of SMP sheets, the pressing force, the heating temperature and the holding time.

(2) The fabricated SMC belt bends in the direction of the shape-memorized round shape of the SME-SMA tape during heating and bends in the direction of the shape-memorized round shape of the SE-SMA tape during cooling. The two-way bending deformation with an angle of 56 degrees was observed.

(3) With respect to the recovery force, the SMC belt was heated and cooled by keeping the initial form in the three-point bending test. Based on the two-way characteristics of the SMC belt, the recovery force increases during heating and decreases during cooling. The simple model was applied to the analysis of the two-way characteristics and confirmed useful in the analysis of the SMC belt.

(4) The characteristics of the SMC depend on the phase transformation temperatures, the volume fractions and the arrangements of the SMA and SMP elements and the rates of heating and cooling. The development and application of the SMC with high function are highly expected.

## References

[1] Funakubo H. (1987). Shape Memory Alloys.

[2] Duerig T.W., Melton K.N., Stockel D., Wayman C.M. (1990). Engineering Aspects of Shape Memory Alloys.

[3] Otsuka K., Waymam C.M. (1998). Shape Memory Materials.

[4] Hayashi S. (1993). Properties and Applications of Polyurethane Series Shape Memory Polymer. Int. Progr. Urethanes.

[5] Tobushi H., Hashimoto T., Ito N., Hayashi S., Yamada E. (1998). Shape Fixity and Shape Recovery in a Film of Shape Memory Polymer of Polyurethane Series. J. Intell. Mater. Syst. Struct..

[6] Tobushi H., Hayashi S., Hoshio K., Ejiri Y. (2008). Shape Recovery and Irrecoverable Strain Control in Polyurethane Shape-Memory Polymer. Sci. Technol. Adv. Mater..

[7] Pieczyska E.A., Gadaj S., Nowacki W.K., Hoshio K., Makino Y., Tobushi H. (2005). Characteristics of Energy Storage and Dissipation in TiNi Shape Memory. Sci. Tech. Adv. Mater..

[8] Tobushi H., Hachisuka T., Hashimoto T., Yamada S. (1998). Cyclic Deformation and Fatigue of a TiNi Shape-Memory Alloy Wire Subjected to Rotating Bending. Trans. ASME J. Eng. Mater. Tech..

[9] Tobushi H., Pieczyska E.A., Ejiri Y., Sakuragi T. (2009). Thermomecanical Properties of Shape-Memory Alloy and Polymer and their Composite. Mech. Adv. Mater. Struct..

[10] Tobushi H., Hayashi S., Hoshio K., Makino Y., Miwa N. (2006). Bending Actuation Characteristics of Shape Memory Composite with SMA and SMP. J. Intell. Mater. Syst. Struct..

[11] Lin P.H., Tobushi H., Tanaka K., Lexcellent C., Ikai A. (1995). Recovery Stress of TiNi Shape Memory Alloy under Constant Strain. Arch. Mech..

